# Rape under the Influence of Ether

**Published:** 1848-03

**Authors:** 


*Rape under the influence of Ether.*—A dentist in France has been lately brought to trial before the Court of Assizes of the Seine, on the charge of having committed criminal assaults on two young women, named Hyacinthe and Henriette, whilst they were in a state of insensibility, caused by the inhalation of the vapor of ether. From the nature of the details which had to be entered into, the trial took place with closed doors; but it transpired that the young women, who had gone to the house of the prisoner for the purpose of having teeth drawn, had been persuaded by him to inhale the vapour of ether, on the ground that it would prevent them from suffering pain; and when he had thrown them into a somnolent state, which, however, they said, did not prevent them from knowing what passed, he committed the criminal assaults complained of. One of the girls declared, that though at the time she knew well what was passing around her, she was totally unable to offer any resistance; and that at the moment the offence was committed, she became unconscious, and remained so for some time.

The prisoner's advocate said that the prisoner totally denied that he was guilty: and the learned gentleman argued that the effects produced by the inhalation of ether on the imagination were such that it was very probable that the girls might have taken their own hallucinations for facts. It, however, appeared that one of the girls, on her return home, had her dress disordered, her hair dishevelled, and was greatly agitated. The jury declared the prisoner guilty, and the Court condemned him to six years' hard labor at the hulks, but without exposure on the pillory. It was also ordered the *syndics* of the prisoners bankruptcy, his arrest having led to bankruptcy, should pay one of the girls, who is under age, a sum of 1,500f. as damages.—*London Med. Gaz.*, Nov. 1847.

